# Restoring the lost heat of the joint: Thermomechanical stimulation as a candidate treatment for early-stage osteoarthritis

**DOI:** 10.1016/j.mbm.2026.100213

**Published:** 2026-09-15

**Authors:** Antoine Reitzel, Deniz Cemre Turgut, Céline Samira Wyss, Vincent Stadelmann, Dominique P. Pioletti

**Affiliations:** a Laboratory of Biomechanical Orthopedics, Institute of Bioengineering, École Polytechnique Fédérale de Lausanne (EPFL), Lausanne, Switzerland; b Department of Research and Development, Schulthess Klinik, Zürich, Switzerland

**Keywords:** Early osteoarthritis, Thermotherapy, Mechanical loading, Cartilage self-heating, Thermo-mechanotransduction, HSP70

## Abstract

Two of the oldest osteoarthritis treatments, local heat and therapeutic exercise, have evolved along separate paths: heat as a symptomatic adjunct and exercise as a functional intervention. Neither intervention alone modifies the disease. This separation reflects clinical tradition, not joint physiology. Articular cartilage dissipates some of the energy absorbed during deformation. At rest, knee cartilage is 32–33 °C and warms toward 37 °C under loading, which enhances matrix production. Physical activity therefore provides two coupled cues: mechanical deformation and the resulting heat. These cues are successive stages of one physical event, not independent therapeutic levers. Critically, this coupling is impaired early in osteoarthritis. Under load, normal-appearing cartilage from an osteoarthritic joint dissipates roughly half as much energy as healthy cartilage. This deficit, which is undetectable by stiffness-based measures, prevents the tissue from heating itself to the optimal temperature range. In vitro, combined thermal and mechanical stimulation elicits stronger chondroinductive and chondroprotective responses than either cue alone, through pathways involving transient receptor potential vanilloid 4 (TRPV4) and TWIK-related potassium 1 (TREK1) channels, calcium signaling, and heat shock protein 70 (HSP70). We propose that early-stage osteoarthritis, when residual matrix and viable chondrocytes remain, offers a therapeutic window in which exogenous heat could restore the lost cue, potentially making exercise a structure-modifying intervention.

## Highlights

• Joint loading heats cartilage: heat and deformation form one coupled physiological cue
• Early osteoarthritic cartilage dissipates 50% less energy before damage is visible
• Combined thermal and mechanical cues outperform either alone via TRPV4/TREK1, HSP70
• Exogenous heating during exercise could restore the lost thermal cue in early OA

## 1 Introduction

Osteoarthritis is among the most prevalent chronic conditions worldwide, with 595 million cases estimated in 2020 and knee cases projected to rise a further 75% by 2050.^1^ Despite this burden, no pharmacological agent has been approved as disease-modifying, and clinical guidelines place exercise, education, and weight management as core first-line care.^2^

Disease-modifying therapies may fail not only because the agents are ineffective, but because they are used too late: once osteoarthritis is diagnosed radiographically, cartilage loss is extensive, and chondrocytes show impaired homeostasis and senescence. Attention has consequently shifted toward early-stage disease as a window in which joint homeostasis might still be restored rather than merely compensated for, an effort now pursued by the Osteoarthritis Research Society International (OARSI) to define the population that could benefit from an intervention that alters the disease trajectory.^3^

The question is therefore no longer when to intervene, but what form that intervention should take. This Perspective advances a biologically grounded approach: controlled local heating combined with controlled mechanical loading, delivered concurrently rather than sequentially. We first review heat as a therapeutic cue, an old clinical practice whose biological rationale has strengthened over the past two decades, and then consider mechanical loading, the field's best-validated intervention. We then argue that these treatments are artificially separated components of a single phenomenon: in healthy cartilage, loading itself generates the thermal signal, exposing chondrocytes to both stimuli simultaneously. We conclude by outlining the requirements for testing this proposition.

## 2 Heat as a therapeutic cue: An old practice with a new rationale

Local heat has been applied to painful joints throughout medical history and remains among the most widely used self-administered treatments for osteoarthritis. Its evidence base is thin: trials show only small effects on movement and swelling, without clear effects on pain or tissue structure. Heat has accordingly been regarded as a comfort measure, with no known biological effect on cartilage.

Two gaps limit interpretation of this evidence. The first is dosimetric: conventional heat interventions use superficial heat, which may not reliably achieve controlled intra-articular warming. In contrast, percutaneous radiofrequency hyperthermia (8 MHz, 200 W, 20 min) raised human knee temperature from 34.4 °C to 39.4 °C while the skin remained near 30 °C, with improvements in pain and function,^4^ although whether it modifies cartilage structure remains unknown.

The second gap is mechanistic. At rest, knee cartilage is 32–33 °C, several degrees below its stimulating temperature range,^5^ so warming the tissue has measurable biological consequences. The best-characterized effector is heat shock protein 70 (HSP70), a stress-responsive chaperone whose heat-induced expression confers chondroprotection in vivo.^6^ Radiofrequency hyperthermia applied to the knees of guinea pigs with spontaneous osteoarthritis also augmented the autophagy proteins Unc-51-like kinase 1 (ULK1) and Beclin1 in mild but not moderate disease, and long-term periodic application reduced cartilage degradation.^7^ This stage dependence argues for targeting early disease.

The thermal window is narrow: proteoglycan metabolism is enhanced at 39–41 °C but suppressed at 43 °C.^8^ The target is therefore mild, transient hyperthermia within the range reached during joint loading (Section 4).

## 3 Mechanical loading: the best-validated intervention, and its limits

Exercise is among the best-validated non-surgical interventions in musculoskeletal medicine, and clinical guidelines designate it as first-line care.^2^ The most recent Cochrane review found low-to moderate-certainty evidence that land-based exercise reduces pain and improves function, with no type outperforming another.^9^ Where cartilage itself was examined, a systematic review found no evidence that exercise damages it, but no consistent structural improvement either.^10^

In vitro, by contrast, chondrocytes respond unambiguously to loading, transducing deformation into transcriptional and biosynthetic responses through mechanosensitive ion channels that convert compression and hydrostatic pressure into calcium signals driving matrix synthesis. Moderate dynamic loading also suppresses the pro-catabolic response to injury, making it both an anabolic and an anti-inflammatory stimulus.^11^

Two factors limit the therapeutic potential of exercise. Biologically, cartilage responds anabolically within a physiological window and catabolically above a strain threshold^11^; this window narrows as tissue quality declines. Clinically, exercise provides predominantly symptomatic benefits, with no demonstrated structural modification.^10^ Thus, loading alone appears insufficient to restore cartilage structure.

## 4 Convergence: the joint generates its own heat

The missing element, as we propose here, is that physiological loading of healthy cartilage does not occur in isolation from thermal stimuli. Cartilage is viscoelastic and biphasic, with high dissipative capacity: under cyclic deformation, part of the strain energy becomes heat, and because cartilage is avascular and removes heat slowly,^12^ this heat accumulates over time. Calorimetry on human cartilage shows that at 1 Hz and 15% strain, the dissipated power predicts a rise toward 37 °C after 40–60 minutes^12^^,^^13^; in vivo measurements confirm that knee temperature climbs from about 32.5 °C at rest to nearly 39 °C during jogging.^5^ Heat is therefore not an adjunct to loading but an intrinsic by-product.

This dissipation has two origins, both related to poroviscoelasticity: flow-dependent viscous drag generated as interstitial fluid is forced through the low-permeability matrix, and flow-independent friction between collagen fibrils and the proteoglycan gel. These mechanisms depend on strain rate.^13^ Degeneration compromises them early: proteoglycan depletion increases permeability, while collagen disruption reduces resistance to fibril sliding. Both mechanisms decline before overt structural damage. Shear testing of human cartilage from arthroplasty donors confirms this finding: normal-appearing tissue from osteoarthritic joints dissipated approximately 50% less energy than healthy cartilage, a deficit undetectable by stiffness-based measures.^14^

Dissipation effect has also been shown directly on cells. Chondro-progenitor cells compressed in scaffolds differing only in dissipation showed upregulated chondrogenic markers at higher dissipation,^15^ while raising culture temperature from 33 °C to 37 °C upregulated SRY-box transcription factor 9 (SOX9) and peroxisome proliferator-activated receptor gamma coactivator 1-alpha (PGC-1α).^13^ In bioreactors, combining strain with a temperature ramp from 32.5 °C to 39 °C had a greater effect on chondrogenic expression than either stimulus alone, with transient receptor potential vanilloid 4 (TRPV4) and TWIK-related potassium 1 (TREK1) acting as mediators.^16^^,^^17^ Similarly, combining hydrostatic pressure with heating produced greater chondroinduction and chondroprotection than either cue alone, with HSP70 serving as a key regulator of the coupled response.^18^ A degenerating joint therefore loses some of its capacity for self-heat before morphological damage appears; exercise then provides only the mechanical component of the stimulus (Fig. 1).

**Fig. 1:**
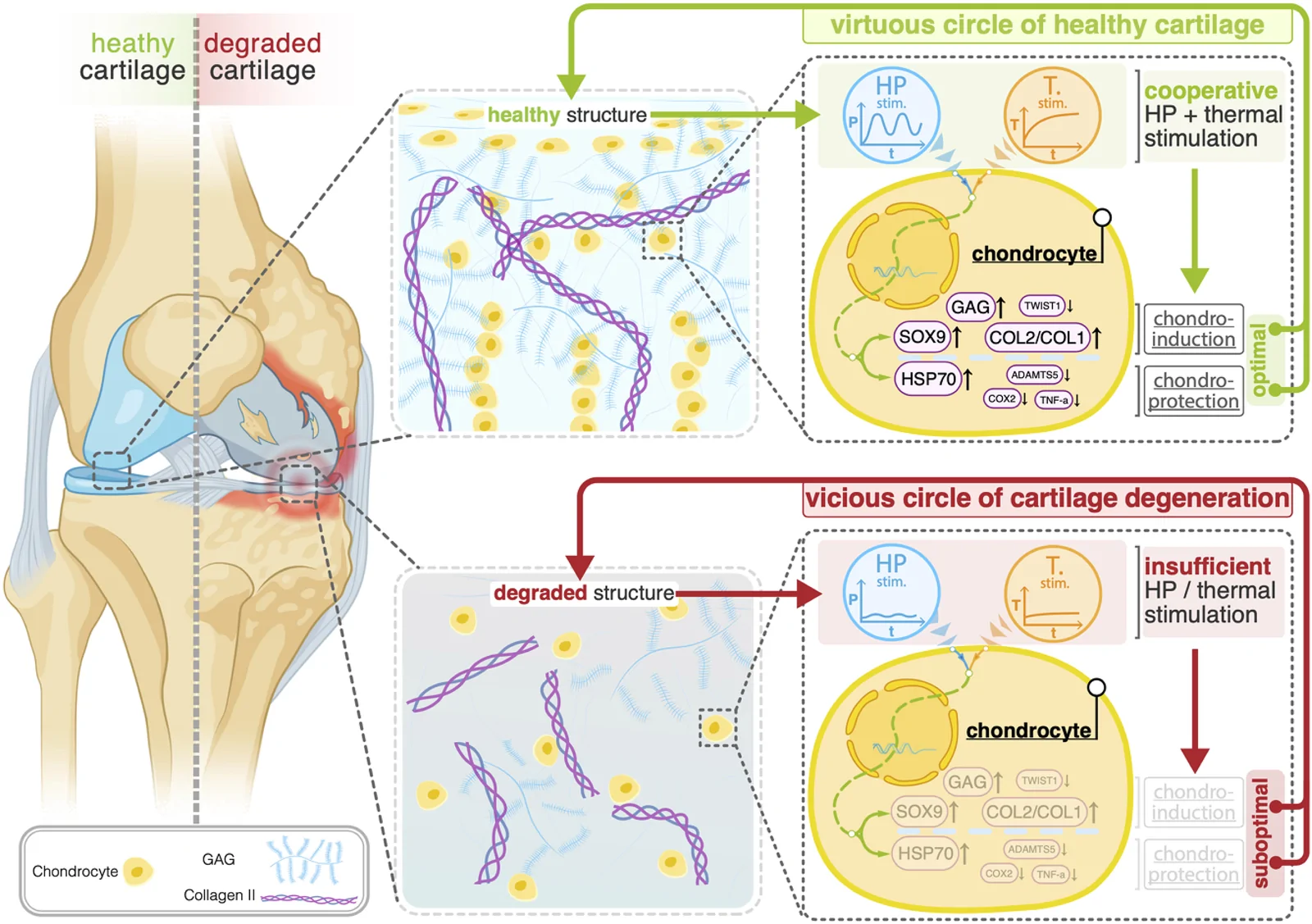
**The virtuous and vicious circles governing cartilage homeostasis under coupled loading and thermal stimulation.** In healthy cartilage (top), an intact proteoglycan–collagen network transmits the two biophysical derivatives of joint loading — deformation and a loading-induced temperature increase — to the resident chondrocytes. Their cooperative action drives maximal chondroinduction (SOX9, COL2/COL1, GAG, TWIST1) and chondroprotection (HSP70), sustaining a virtuous circle of matrix maintenance. In degenerated cartilage (bottom), structural breakdown of the matrix diminishes both cues, so the chondrocyte receives insufficient loading and thermal stimulation; chondroinduction and chondroprotection fail while catabolic and inflammatory mediators (ADAMTS5, COX2, TNF-α) rise, perpetuating a self-reinforcing vicious circle of cartilage degradation. The early loss of the loading-induced thermal cue, which the degenerating tissue can no longer generate through energy dissipation, is thus positioned as an initiating and reinforcing event in this cascade. Adapted from Y. Guo, EPFL doctoral thesis no. 10509.

## 5 What a thermomechanical treatment would look like

No combined thermomechanical treatment for osteoarthritis currently exists in clinical practice; heat and exercise are delivered separately, and the available evidence concerns their individual components. What follows is therefore a specification of what such a treatment would require, not a description of an available therapy. Indeed, translating combined mechanical and thermal loading into a therapeutic intervention requires specifying four parameters: thermal target, delivery method, mechanical dose, and temporal relationship.

The thermal target should be a transient increase in intra-articular temperature to approximately 38–40 °C, mimicking rather than exceeding physiological self-heating. This target is deliberately conservative relative to the 41–43 °C range explored in hyperthermia studies and remains well below the threshold for collagen damage. Importantly, this temperature range has already been achieved in the human knee using 8 MHz radiofrequency hyperthermia.^4^

Delivery should use a deep-heating modality. Superficial approaches such as hot packs and paraffin confine the temperature rise to skin and subcutaneous tissue.^19^ Microwave diathermy at 433.92 MHz improved symptoms in a randomised trial, though without intra-articular temperature measurement.^20^ Percutaneous radiofrequency hyperthermia raises intra-articular temperature into the target range,^4^ and a resonant-cavity applicator has been shown to warm the cartilage region without heating the overlying tissue, so far only in healthy volunteers.^19^ No modality has yet been validated for delivering a controlled intra-articular dose during exercise, which remains the principal engineering obstacle.

The mechanical dose should remain within the established physiological anabolic window rather than approach its limits, given evidence of a strain threshold beyond which loading becomes catabolic.^11^ In practice, this favors supervised, moderate, cyclic weight-bearing activity as recommended in existing guidelines,^2^^,^^9^ rather than more intensive loading.

The temporal relationship marks the proposal's clearest departure from current practice, and is the aspect least informed by clinical evidence. In current practice, heat is applied before exercise as a preparatory measure. The mechanistic argument instead favors concurrent delivery of the two cues, reflecting both their physiological co-occurrence and the in vitro synergy observed under simultaneous application 16, 17, 18.

## 6 Conclusions

Heat and mechanical loading have long been used to treat osteoarthritis in parallel but largely independently. Cartilage physics indicates that this separation has no basis in joint physiology: loading viscoelastic tissue generates heat, and the healthy joint therefore delivers both cues together. Degeneration disrupts this coupling early, potentially explaining why exercise modifies symptoms more readily than structure. This suggests a testable intervention: applying exogenous heat during mechanical loading in patients whose cartilage retains viable cells. Testing this proposition will first require human osteoarthritic explants stratified by degeneration stage, followed by in vivo work comparing the combined treatment with loading alone. Reliable in vivo thermal dosimetry will also be essential.

## CRediT authorship contribution statement

**Antoine Reitzel:** Writing – review & editing. **Deniz Cemre Turgut:** Writing – review & editing. **Céline Samira Wyss:** Writing – review & editing. **Vincent Stadelmann:** Writing – review & editing. **Dominique P. Pioletti:** Writing – original draft.

## Declaration of generative AI and AI-assisted technologies in the writing process

During the preparation of this work, the authors used Claude (Anthropic) to help structure the argument, draft and revise sections of text under author direction, and synthesize the literature supplied by the authors. After using this tool, the authors reviewed and edited the content as needed, verified every cited reference against the original publication, and take full responsibility for the content of the publication.

## Ethical approval

This study does not contain any studies with human or animal subjects performed by any of the authors.

## Declaration of competing interest

The authors declare that they have no known competing financial interests or personal relationships that could have appeared to influence the work reported in this paper.

## References

[1] GBD 2021 Osteoarthritis Collaborators (2023). Global, regional, and national burden of osteoarthritis, 1990–2020 and projections to 2050: a systematic analysis for the Global Burden of Disease Study 2021. Lancet Rheumatol.

[2] Bannuru R.R., Osani M.C., Vaysbrot E.E. (2019). OARSI guidelines for the non-surgical management of knee, hip, and polyarticular osteoarthritis. Osteoarthr Cartil.

[3] Mahmoudian A., Lohmander L.S., Mobasheri A., Englund M., Luyten F.P. (2021). Early-stage symptomatic osteoarthritis of the knee — time for action. Nat Rev Rheumatol.

[4] Takahashi K., Kurosaki H., Hashimoto S., Takenouchi K., Kamada T., Nakamura H. (2011). The effects of radiofrequency hyperthermia on pain and function in patients with knee osteoarthritis: a preliminary report. J Orthop Sci.

[5] Becher C., Springer J., Feil S., Cerulli G., Paessler H.H. (2008). Intra-articular temperatures of the knee in sports – an in-vivo study of jogging and alpine skiing. BMC Muscoskelet Disord.

[6] Tonomura H., Takahashi K.A., Mazda O. (2008). Effects of heat stimulation via microwave applicator on cartilage matrix gene and HSP70 expression in the rabbit knee joint. J Orthop Res.

[7] Takahashi K., Nakamura H., Ozawa H. (2018). Effectiveness of radiofrequency hyperthermia for treating cartilage in Guinea pigs with primary osteoarthritis. Cartilage.

[8] Hiraoka N., Takahashi K.A., Arai Y. (2009). Hyperthermia for the treatment of articular cartilage with osteoarthritis. Int J Hyperther.

[9] Lawford B.J., Hall M., Hinman R.S. (2024). Exercise for osteoarthritis of the knee. Cochrane Database Syst Rev.

[10] Bricca A., Juhl C.B., Steultjens M., Wirth W., Roos E.M. (2019). Impact of exercise on articular cartilage in people at risk of, or with established, knee osteoarthritis: a systematic review of randomised controlled trials. Br J Sports Med.

[11] Li Y., Frank E.H., Wang Y., Chubinskaya S., Huang H.-H., Grodzinsky A.J. (2013). Moderate dynamic compression inhibits pro-catabolic response of cartilage to mechanical injury, tumor necrosis factor-α and interleukin-6, but accentuates degradation above a strain threshold. Osteoarthr Cartil.

[12] Abdel-Sayed P., Moghadam M.N., Salomir R., Tchernin D., Pioletti D.P. (2014). Intrinsic viscoelasticity increases temperature in knee cartilage under physiological loading. J Mech Behav Biomed Mater.

[13] Abdel-Sayed P., Vogel A., Moghadam M.N., Pioletti D.P. (2013). Cartilage self-heating contributes to chondrogenic expression. Eur Cell Mater.

[14] Maier F., Lewis C.G., Pierce D.M. (2019). The evolving large-strain shear responses of progressively osteoarthritic human cartilage. Osteoarthr Cartil.

[15] Abdel-Sayed P., Darwiche S.E., Kettenberger U., Pioletti D.P. (2014). The role of energy dissipation of polymeric scaffolds in the mechanobiological modulation of chondrogenic expression. Biomaterials.

[16] Nasrollahzadeh N., Karami P., Wang J. (2022). Temperature evolution following joint loading promotes chondrogenesis by synergistic cues via calcium signaling. eLife.

[17] Stampoultzis T., Guo Y., Nasrollahzadeh N., Karami P., Pioletti D.P. (2023). Mimicking loading-induced cartilage self-heating in vitro promotes matrix formation in chondrocyte-laden constructs with different mechanical properties. ACS Biomater Sci Eng.

[18] Guo Y., Stampoultzis T., Karami P., Nasrollahzadeh N., Rana V.K., Pioletti D.P. (2024). HSP70 — a key regulator in chondrocyte homeostasis under naturally coupled hydrostatic pressure-thermal stimuli. Osteoarthr Cartil.

[19] Shindo Y., Takahashi K., Ikuta F., Iseki Y., Harada K., Kato K. (2020). Deep heat therapy system with resonant cavity applicator for articular cartilage in knee osteoarthritis. J Phys Ther Sci.

[20] Giombini A., Di Cesare A., Di Cesare M., Ripani M., Maffulli N. (2011). Localized hyperthermia induced by microwave diathermy in osteoarthritis of the knee: a randomized placebo-controlled double-blind clinical trial. Knee Surg Sports Traumatol Arthrosc.

